# Carcinoid Tumor of the Common Bile Duct

**DOI:** 10.1155/1996/51493

**Published:** 1996

**Authors:** Doron Kopelman, Moshe Schein, Hedviga Kerner, Hany Bahuss, Moshe Hashmonai

**Affiliations:** Department of Surgery B and the Department of Pathology Rambam Medical Center and the Technion- Israel Institute of Technology Haifa 31096 Israel

## Abstract

A case of a primary carcinoid tumor of the common bile duct is presented. Diagnostic and therapeutic uncertainties of this extremely rare cause of jaundice are discussed.


*HPB Surgery*, 1996, Vol.10, pp.41–43
Reprints available directly from the publisher
Photocopying permitted by license only

© 1996 OPA (Overseas Publishers Association)
Amsterdam B.V. Published in The Netherlands
by Harwood Academic Publishers GmbH
Printed in Malaysia

### CASE REPORT
### Carcinoid Tumor of the Common Bile Duct

DORON KOPELMAN, MOSHE SCHEIN, HEDVIGA KERNER,
HANY BAHUSS and MOSHE HASHMONAI

Department of Surgery B and the Department of Pathology*, Rambam Medical Center and the
Technion- Israel Institute of Technology, Haifa, Israel

*(Received 24 October 1993)*

A case of a primary carcinoid tumor of the common bile duct is presented. Diagnostic and therapeutic uncertainties of this extremely rare cause of jaundice are discussed.

KEY WORDS: Carcinoid tumor biliary tract obstructive jaundice pancreaticoduodenectomy

### INTRODUCTION

Argentaffin, carcinoid tumor producing cells, are present in all parts of the gastrointestinal tract including the biliary system[1]. The most frequent gastrointestinal primary sites for the development of carcinoid tumors are the small bowel, rectum, appendix and stomach[2]. Carcinoid tumors of the biliary tract, on the other hand, are sporadically reported and arise mostly in the gallbladder[3,4], whereas such tumors of the main bile ducts are extremely rare and have been Reported only on four occasions[4–7].

We present herein another case of a carcinoid tumor of the common bile duct presenting with obstructive jaundice. Diagnostic and therapeutic uncertainties are emphasized.

### *Case report*

A 44 year-old male patient was admitted with resolving obstructive jaundice. Previous ambulatory ultrasonography has demonstrated a mild distention of the intra- and extra- hepatic bile ducts and a dilated gallbladder. This has been followed by an endoscopic retrograde cholangiography (ERCP), which disclosed a fusiform stricture of the distal common bile duct; it has been dilated and a 10 F stent was inserted. There were no positive findings on physical examination except for residual jaundice. After the jaundice subsided the patient underwent an explorative laparotomy. A 0.5 cm' lesion was found on the anterior surface of the left hepatic lobe and was fully excised; frozen section reported an anaplastic carcinoma. The extra- hepatic bile ducts, gallbladder and pancreas appeared macroscopically normal. The condition was assessed as a 'metastatic carcinoma of presumably biliary or pancreatic origin' and a palliative choledochoduodenostomy was undertaken. Frozen section of the choeledochotomy's normally appearing edges revealed an anaplastic carcinoma as well.

The postoperative course was uneventful. Pathological examination of the paraffin sections showed a small (0.5 cm') metastatic carcinoid tumor in the liver. The tumor cells were arranged in groups of small uniform cells without conspicuous atypical changes (Fig. 1). The diagnosis of carcinoid tumor was supported by a positive immunohistochemical stain for chromogranin. The tumor infiltrating the wall of the common bile duct showed the same small uniform groups of cells (Figure 2) and perineural infiltration was seen. This tumor was interpreted as a carcinoid of the bile duct.

*Correspondence to*: Moshe Hashmonai, MD, FACS, Department of Surgery B, Rambam Medical Center, P.O. Box 9602, Haifa 31096, Israel

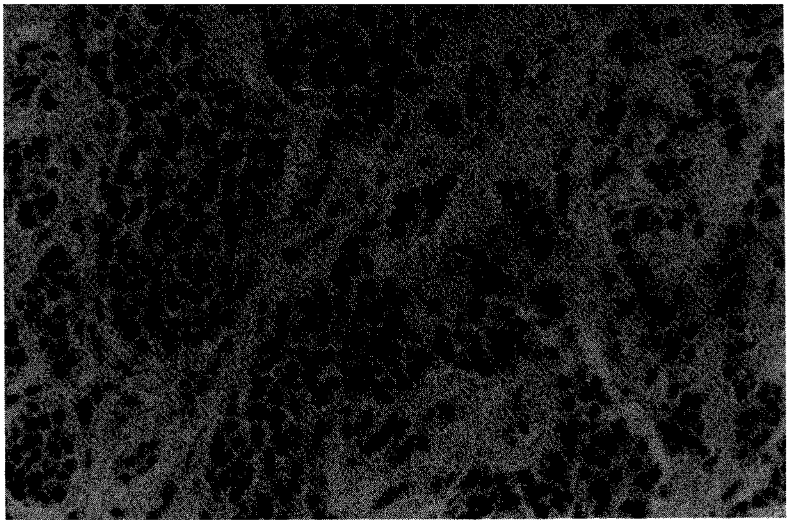

**Figure 1** The metastatic carcinoid tumor in the liver (H & E, × 300).

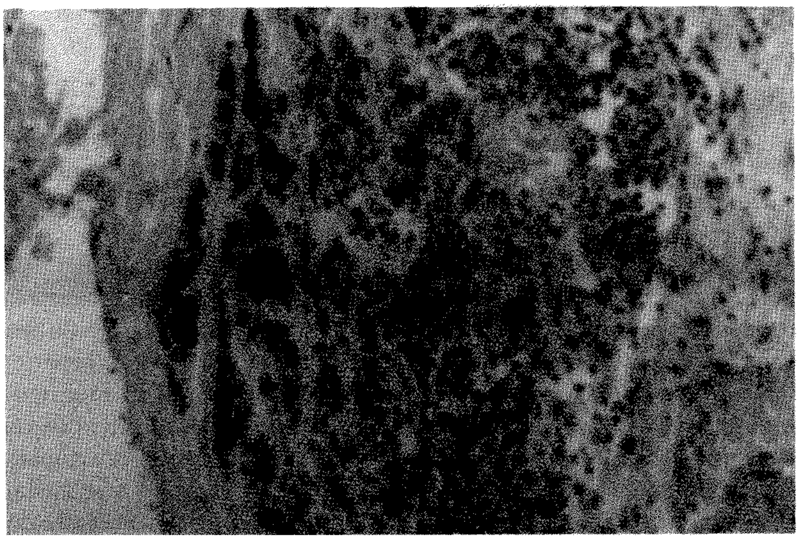

**Figure 2** The carcinoid tumor in the wall of the common bile duct (H & E, × 300).

The literature was searched for therapeutic guidelines to no avail. Taking into consideration the minimal hepatic involvement and the patient's young age it was decided to proceed with a pancreatico-duodenectomy in order to eliminate the primary tumor which was suspected still to exist at the site of the choledocoduodenostomy. The latter was excised at a pylorus-preserving Whipple's procedure during which the liver and regional lymph-nodes appeared normal. Careful pathological assessment of the resected specimen failed to find any evidence of residual carcinoid tumor. All excised lymph nodes were free of tumor. Presently, 18 months after the operation the patient is well.

### DISCUSSION

The extreme rarity of carcinoid tumor causing obstructive jaundice makes its pre-operative diagnosis very unlikely. This case of a carcinoid tumor of the common bile duct is the fifth reported in the English literature. Also the four previously described patients presented with obstructive jaundice; one was diagnosed at autopsy[4], another at operation[6] and the details of the remaining two were not mentioned[5,7].

In this respect the situation is similar to that occurring with other uncommon benign tumors and pseudotumors or sarcoma of the bile ducts, all conditions clinically mimicking the commonly occurring cholangiocarcinoma[8]. Obviously, routine pre-operative investigations of the jaundiced patient such as ultrasound, CT, and ERCP are not able to pinpoint the correct diagnosis. As reflected in this case and emphasized by others[2–4], the primary carcinoids of the gastrointestinal tract may be of a small diameter and yet metastases may be found. Perhaps, needle aspiration of a mass, if visualized on CT or brushing cytology at ERCP, could achieve pre-operative diagnosis. Also accurate pre- and intra-operative histological diagnosis may be problematic because the differentiation between carcinoid tumors and undifferentiated carcinoma is very difficult (Figures 1 & 2), particularly on frozen section specimen.

This case exemplifies the dangers of "blind" stenting of the obstructed bile duct, without histological diagnosis, in the jaundice patient labeled (following CT and ERCP examinations) as suffering from 'malignant obstruction with metastatic disease'. Such approach may deny definitive treatment in the occasionally curable patient while exposing him to the prolong morbidity of non-operative palliation.

The paucity of published experience with carcinoid tumor of the bile duct makes therapeutic recommendations not more than a learned guess. However, experience with carcinoids elsewhere in the gastrointestinal tract, which suggest the advisability of aggressive resection or debulking may be applied [9,10]. Primary tumors should be removed and if deemed unresectable debulking should be attempted. Likewise, solitary liver metastases should be resected. The role of pancreaticoduodenectomy as performed in this patient, remains unproven. Interestingly, no evidence of tumor was found in the specimen of our Whipple's procedure attesting to the minute size of the primary carcinoid causing jaundice in this patient, and the possibility that it was completely destroyed during biopsy and choledoduodenostomy.

### REFERENCES

1. Christie A. C. (1954) Three cases illustrating presence of Argentaffin (Kultschitsky) cells in human gallbladder. *J Clin Pathol.* **7**: 318–321.
2. Thompson G. B, van Heerden J. A, Martin J. K, Schutt A. J, Ilstrup D. M, Garney J. A. (1985) Carcinoid tumors of the gastrointestinal tract: presentation, management and prognosis. *Surgery* **98**: 1054–1062.
3. Shiffman M. A, Juler G. (1963) Carcinoid of the biliary tract. *Arch Surg* **89**: 1113–1115.
4. Begdahl L. (1976) Carcinoid tumours of the biliary tract. *Aust NZ J Surg* **46**: 136–138.
5. Davies AJ. (1959) Carcinoid tumours (Argentaffinomata). *Ann Roy Coll Surg* **25**: 277–280.
6. Little J. M, Gibson A. A. M, Kay A.W. (1968) Primary common bile-duct carcinoid. *Br J Surg* **55**:147–149.
7. Godwin J. D. (1975) Carcinoid tumors: An analysis of 2837 cases. *Cancer* **36**: 560–569.
8. Beazley R. M, Blumgart L. H. (1988) Benign tumours and pseudotumours of the biliary tract. In Blumgart LH, ed, Surgery of the liver and biliary tract. Churchill Livingstone, London, p 807–818.
9. Soreide O, Berstad T, Bakka A *et al.* (1992) Surgical treatment as a principle in patients with advanced abdominal carcinoid tumors. *Surgery*; **111**: 48–54.
10. Wareing T. H, Sawyers J. L. (1983) Carcinoids and the carcinoid syndrome. *Am J Surg* **145**: 769–772.

